# Electrical Impedance Spectroscopy for Electro-Mechanical Characterization of Conductive Fabrics

**DOI:** 10.3390/s140609738

**Published:** 2014-06-02

**Authors:** Tushar Kanti Bera, Youssoufa Mohamadou, Kyounghun Lee, Hun Wi, Tong In Oh, Eung Je Woo, Manuchehr Soleimani, Jin Keun Seo

**Affiliations:** 1 Department of Computational Science and Engineering, Yonsei University, Seoul 120-749, Korea; E-Mails: tkbera77@gmail.com (T.K.B.); imlkh84@gmail.com (K.L.); seoj@yonsei.ac.kr (J.K.S.); 2 Department of Biomedical Engineering, Kyung Hee University, Yongin 446-701, Korea; E-Mails: usufcom@hotmail.com (Y.M.); mogly1126@nate.com (H.W.); ejwoo@khu.ac.kr (E.J.W.); 3 Department of Electronic and Electrical Engineering, University of Bath, Bath BA2 7AY, UK; E-Mail: ms350@bath.ac.uk

**Keywords:** electrical impedance spectroscopy (EIS), conductive fabric, tension, compression, electromechanical property

## Abstract

When we use a conductive fabric as a pressure sensor, it is necessary to quantitatively understand its electromechanical property related with the applied pressure. We investigated electromechanical properties of three different conductive fabrics using the electrical impedance spectroscopy (EIS). We found that their electrical impedance spectra depend not only on the electrical properties of the conductive yarns, but also on their weaving structures. When we apply a mechanical tension or compression, there occur structural deformations in the conductive fabrics altering their apparent electrical impedance spectra. For a stretchable conductive fabric, the impedance magnitude increased or decreased under tension or compression, respectively. For an almost non-stretchable conductive fabric, both tension and compression resulted in decreased impedance values since the applied tension failed to elongate the fabric. To measure both tension and compression separately, it is desirable to use a stretchable conductive fabric. For any conductive fabric chosen as a pressure-sensing material, its resistivity under no loading conditions must be carefully chosen since it determines a measurable range of the impedance values subject to different amounts of loadings. We suggest the EIS method to characterize the electromechanical property of a conductive fabric in designing a thin and flexible fabric pressure sensor.

## 1. Introduction

Conductive fabrics [1–10] have been mainly used for electrostatic discharge, shielding, signal or power transmission, and heating. To take advantage of their flexibility, durability, and washability, they are finding new applications as sensors in biomedicine and robotics: smart textiles [1–5], bioimpedance monitoring [6,7], electrodes [8–10], pressures sensors [11–14], textile electronics [15,16], and wearable sensors [17–21].

In this paper, we focus on the application of conductive fabrics as a thin and flexible pressure sensor. Each conductive fabric has a distinct internal structure and composition that determine its electromechanical behavior. Under compression or tension, there occurs a mechanical deformation in the conductive fabric, and this should result in some changes in its electrical properties. A conductive fabric exhibits its own electromechanical properties since a mechanical deformation alters its electrical properties. Knowledge of the electromechanical properties is necessary to design a pressure sensor using the flexible conductive fabric. There are several studies about the mechanical stress–strain analyses of woven textile composites under compression or tension [22,23] and the electrical properties of conductive fabrics [24–29]. However, there is little previous work on the electromechanical characteristics of conductive fabrics.

To explore the electromechanical responses of conductive fabrics for their applications in pressure sensing, we chose three types of conductive fabrics to be studied by the electrical impedance spectroscopy (EIS) method [30–32] under compression and tension. To our knowledge, there are no previous studies that investigate the electromechanical properties of a conductive fabric using the EIS method.

We plan to develop a pressure distribution imaging system using a conductive fabric. Since we intend to produce images of the fabric's electrical properties, in this paper, we study how the electrical properties are affected by mechanical compression and tension. When we apply a mechanical pressure on a conductive fabric, a structural deformation is induced to produce a measurable change in its electrical impedance. We should investigate this change in terms of its magnitude, phase, direction, and also frequency dependence, depending on the applied load.

After describing the details of the chosen materials, we will explain the EIS methods used to characterize the conductive fabrics. Using numerical simulation and experimental measurement methods, we will evaluate the pressure-induced impedance changes at different frequencies. We will analyze the electromechanical behavior of the chosen conductive fabrics through the measured impedance changes over a chosen frequency range.

## 2. Materials and Methods

### 2.1. Conductive Fabrics

Conductive yarns are made from conductive and semi-conductive materials that can be blended in various ways such as coating or twisting [33–38]. A non-conductive or semi-conductive substrate such as cotton, polyester, or nylon is either coated or embedded with electrically conductive elements such as carbon, nickel, copper, gold, silver, or titanium. Conductive fabrics are usually manufactured using carbon- or metal-coated yarns. The electromechanical property of a conductive fabric is determined by the material properties of the yarns and the weaving or knitting methods to construct the interlacing structure of the fabric. The particular weave of a fabric also defines the characteristics such as the flexibility, sheen, texture, and appearance.

**Figure 1.**
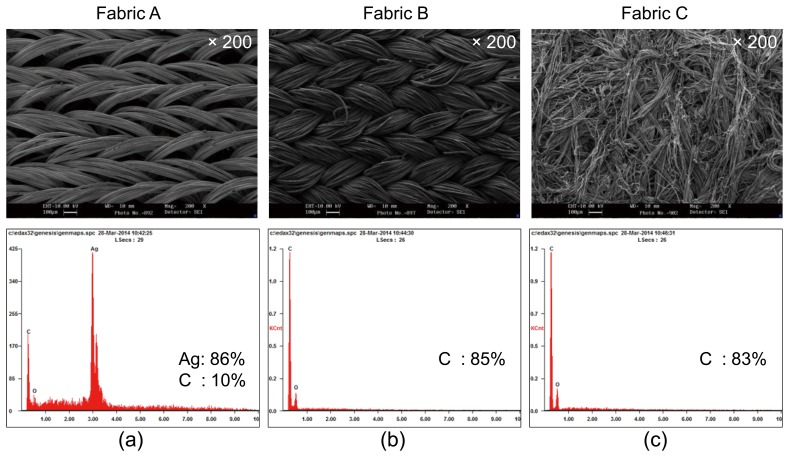
SEM images (×200) and EDS microanalyses of three conductive fabrics. Fabric A was plated with silver. Fabrics B and C were coated with carbon. Figures in the upper and lower rows are the SEM images and the EDS microanalysis results, respectively.

We chose three commercially available conductive fabrics with different compositions and structures. Fabric A (Figure 1a) is a silver plated nylon-based elastic fiber fabric with Lycra-like stretch called the stretch conductive fabric (Cat. #321, Less EMF Inc., Latham, NY, USA) [39]. As shown in its SEM image, its structure is not tight and there are large air gaps among the fibers to allow high elasticity. With its resistivity of 0.5 Ω/m2, it is the most conductive. It has been used for antibacterial wound and burn dressings and also as electromagnetic shielding [39]. The weight percentages and atomic percentages of silver and carbon for fabric A were 86.39 Wt%/42.76 At% and 10.69 Wt%/42.52 At%, respectively.

Fabric B (Figure 1b) is a knitted nylon/Spandex coated with carbon (EeonTex™ LR-SL-PA-10E5, Eeonyx Corp., Pinole, CA, USA) [40]. Its SEM image shows a tightly knitted structure. It can be stretched a little in the longitudinal direction. It is the least conductive, with a resistivity of 10^5^ Ωm^2^. It has 85% carbon.

Fabric C (Figure 1c) is a nonwoven microfiber coated with carbon (NW170-SL-PA-1500, Eeonyx Corp., Pinole, CA, USA) [41]. Since the microfibers are strongly attached each other in a non-uniform structure, it is almost non-stretchable. It is moderately conductive, with a resistivity of 1500 Ω/m^2^. It has 83% carbon. Figure 1 shows the Energy Dispersive Spectrometry (EDS) results together with the SEM images of the fabrics.

### 2.2. Basics for EIS of Conductive Fabrics

The electrical impedance is a complex quantity consisting of resistance and reactance in its real and imaginary parts, respectively. The resistance is determined by the conductivity, shape, and size of a sample. The reactance is determined by its permittivity, shape, and size. The conductivity is a material property that includes the effects of the concentrations of mobile charges and their mobility. The permittivity is also a material property that includes the effects of the dielectric polarization and capacitances among microscopic conductive surfaces. The fabrication methods of a conductive fabric, including its composition, weaving, coating, and density, should affect these properties. For the design of a fabric sensor, we can choose its shape and size depending on a specific application.

A conductive fabric appears to be resistive under an applied DC current. For an AC current, its behavior becomes complex with both resistive and reactive terms since the interactions of the conductive yarns and air gaps produce apparent capacitances. A stretchable conductive fabric shows a more interesting behavior. When it is compressed or pulled, there occur structural deformations of both the yarns and weaves, which result in measurable changes of its apparent electrical impedance value, therefore, we can investigate the electromechanical behavior of a fabric subject to applied pressures through its measured electrical impedance values at multiple frequencies.

In conventional electrical impedance spectroscopy (EIS), we inject a sinusoidal current with constant amplitude and phase and measure an induced voltage in a chosen frequency range. Applying Ohm's law, we can obtain a complex impedance spectrum, *Z*(*ω*) where *ω* is the angular frequency.

In this paper, we performed EIS measurements of the chosen fabrics under varying amounts of applied pressures. Therefore, we will denote the measured impedance spectrum as *Z*(*ω*,*p*) where *p* is the applied pressure. It presents the electromechanical property of the fabric including the effects of its internal structural changes subject to the applied pressure. We denote the real and imaginary parts of *Z*(*ω*,*p*) as *R_z_* and *X_z_*, respectively. Both in numerical simulations and experiments, we prepared all the fabrics in the same shape and size before we applied any loading.

### 2.3. EIS Numerical Simulations of Conductive Fabrics

**Figure 2.**
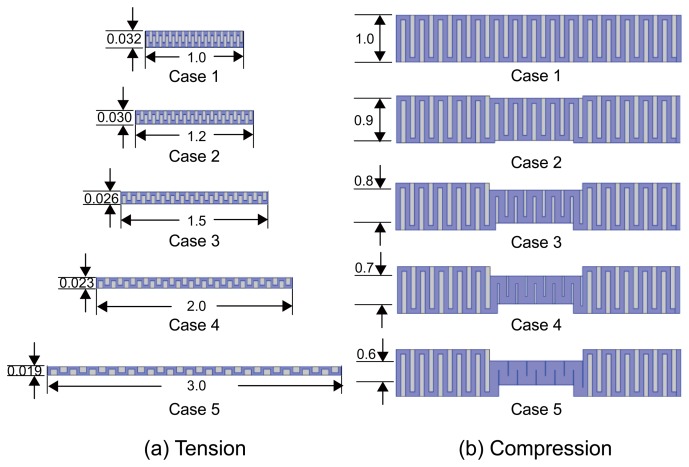
Numerical models of a conductive fabric under tension and compression. The blue and grey regions denote the conductive yarns and air gaps, respectively. (**a**) Models of the conductive fabric subject to different amounts of tension at the edges. (**b**) Models of the conductive fabric subject to compressive forces at the middle.

To provide a theoretical background about the electromechanical property of a conductive fabric, we designed two numerical simulations of the EIS measurements with tension and compression as shown in Figure 2. We assumed an elastic fabric weaved in a uniform structure to analyze the effects of its structural deformation and air volume changes among conductive yarns. We used COMSOL (COMSOL Inc., Burlington, MA, USA) to numerically solve the partial differential equation for the models shown in Figure 2.

First, we considered the numerical model of a conductive fabric under tensile forces. The width to thickness ratio of the two-dimensional model without any force was 1:0.032. We applied four different tensile forces to extend its length by 1.2, 1.5, 2.0, and 3.0 times compared with the case of no loading. We decreased the width by 0.9, 0.8, 0.7, and 0.6, respectively. In the blue region of the yarns, we set the conductivity and relative permittivity values as 0.01 S/m and 1.5, respectively. For the grey region presenting air gaps between yarns, we assumed the conductivity of 10^−10^ S/m and a relative permittivity of 1. In the case of a tensile force, the fabric fibers are stretched and the diameters of the yarns become thinner. As the contact areas among the fibers are decreased, we can expect the impedance to increase.

In the second simulation, we applied a compressive force at the center region of a two-dimensional fabric model (33% of the total length). We decreased the thickness of the compressed region depending on the applied force. With the relative thickness without loading set as 1, the thickness was decreased by 0.9, 0.8, 0.7, and 0.6, respectively, for the same amounts of loadings used in the first simulation. For the blue region of the yarns, we use the conductivity of 0.05 S/m and the relative permittivity of 1.5. The air gaps shown in grey color had the same conditions as in the first simulation.

Under compression, the fabric fibers are flattened with subsequent increases in fiber diameters. This makes the fibers come closer each other. Thus, the area of the air gaps decreases and the contact area among the fibers increase. From these, we can expect the impedance to decrease.

### 2.4. EIS System

To measure the impedance spectrum of each fabric under tension and compression, we used a commercial electrical impedance analyzer (Solartron 1260, AMETEK Inc., Berwyn, PA, USA). Since the performance of the impedance analyzer deteriorates beyond 1 MHz as described in its datasheet [42], we collected the impedance data below 1 MHz.

Noting that the impedance analyzer produces erroneous results for very small and also very large values of the load impedance, we used the compensation method described in Mohamadou *et al.* [43] to improve the measurement accuracy. In summary, we used a set of small and large loads with known impedance values and obtained their impedance spectra using the analyzer. This allowed us to find the system transfer functions of the analyzer. Using this information, we could compensate the measured spectra of the fabrics for better accuracy when their impedance values were small or large.

**Figure 3.**
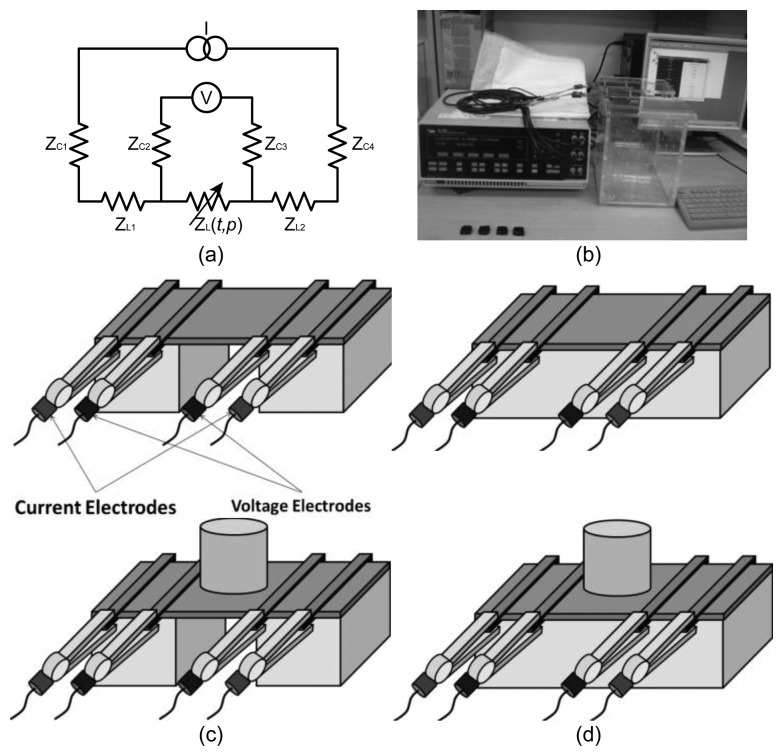
EIS measurements of conductive fabrics under tension and compression. (**a**) Four-electrode method for impedance measurements. (**b**) Measurement setup using the Solartron 1260 impedance analyzer. (**c**) EIS measurement setup for applied tension using acrylic supports. (**d**) EIS measurement setup for applied compression.

As shown in Figure 3a, we adopted the four-electrode method where the current was injected through the outer pair of the electrodes and the voltage was measured between the inner pair of electrodes [44]. Using a constant current source and a differential voltage amplifier with a very high input impedance, we could get rid of the undesirable effects of the contact impedance between the electrode and the sample and minimize the measurement errors.

### 2.5. EIS Experiments of Conductive Fabrics

We prepared the samples of the fabrics A, B, and C as sheets of 150 × 50 mm^2^. Their thicknesses were 0.4, 0.4, and 0.6 mm, respectively. Since the dimensions of the samples were fixed, we compared the measured impedance values instead of extracting their conductivity and permittivity values which are independent of the geometrical factors.

We used two different acrylic scaffolds to hang the sample and connect them to four long stainless steel electrodes (Figure 3b). One was to measure the impedance spectrum subject to tension (Figure 3c) and the other was for the case of compression (Figure 3d). Four rectangular stainless steel rods and acrylic screws tightly hold the fabric samples. Tension or compression was applied to the fabric sample by using insulating weights of 0, 25, 50, 75, 100, 125, and 150 gram-force placed on the center of the sample. We collected the impedance data at 14 frequencies between 50 Hz and 1 MHz to plot its spectrum.

## 3. Results

### 3.1. Numerical Simulation Results

**Figure 4.**
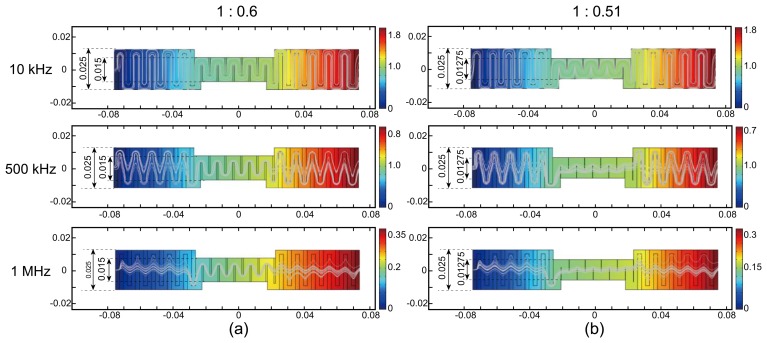
Computed electric potential distributions and current streamlines inside the fabric under two different amounts of compression. The color bars denote the electric potential subject to the injected current from the right to left direction. The curved lines are current streamlines. (a) and (b) show the electric potential distributions and current streamlines, respectively, at 10, 500, and 1000 kHz subject to two different amounts of compression (1:0.6 and 1:0.51).

Figures 4a,b show the results of two different amounts of compression expressed as thickness ratios of 1:0.6 and 1:0.51, respectively. We computed the electric potential distribution and current streamlines inside the fabric. As we increase the frequency of the injected current, the electric current streamlines became straighter due to the decreased reactance terms at high frequency. This phenomenon was more distinct for higher compression, as seen in Figure 4b. For the results in Figure 4, the conductivity and relative permittivity of the simulated fabric were 0.05 S/m and 1.5, respectively. We assumed the air conductivity of 10^−10^ S/m. We could observe similar results for all five cases shown in Figure 2. For tension, the current streamlines did not change much at frequencies below 1 MHz.

**Figure 5.**
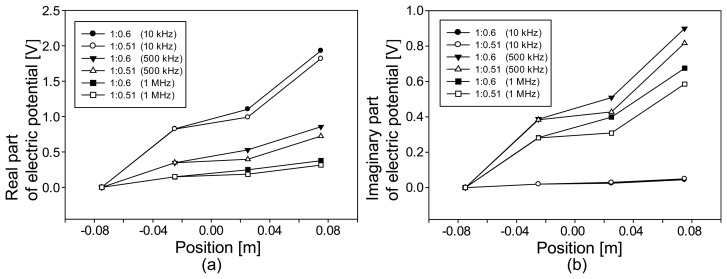
Changes of the computed electric potentials along the middle horizontal line: (**a**) real and (**b**) imaginary parts.

Figures 5a,b display the profiles of the real and imaginary parts, respectively, of the computed electric potentials at 10, 500, and 1000 kHz along the horizontal line at the middle of the fabric for two different amounts of compression shown in Figure 4. Figure 5a shows that the larger amount of compression made the compressed region more conductive and resulted in a smaller slope. In Figure 5b, we can observe that the larger amount of compression produced smaller reactance values, that is, larger capacitance values. For all five cases in Figure 2, we could obtain similar results.

For tension, the values of the real part were smaller than those in Figure 5a. Since the fabric was elongated uniformly under tension, the voltage changed linearly throughout the entire fabric along the horizontal direction. The values of the imaginary part also changed linearly for the same reason.

**Figure 6.**
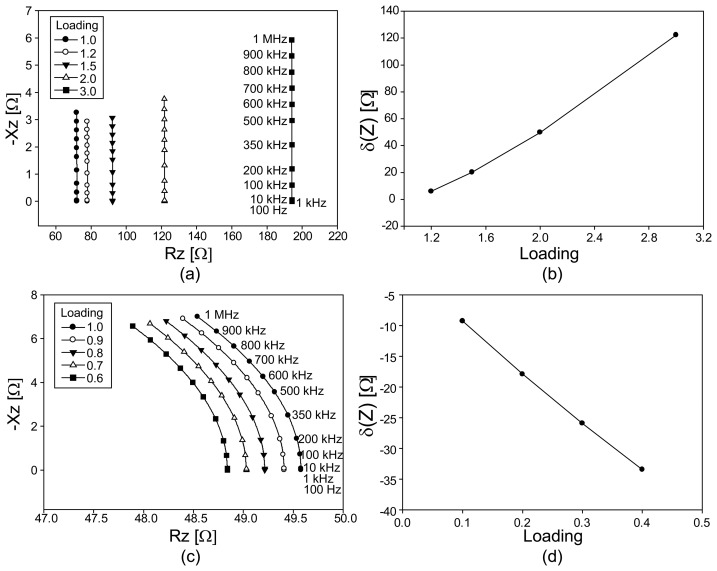
Simulation results of impedance spectra of conductive fabrics under tension and compression. (**a**) and (**c**) are the Argand diagrams. (**b**) and (**d**) show changes in the magnitude of the impedance for different amounts of loadings.

Figure 6 summarizes the numerical simulation results of the computed impedance spectra. Figures 6a,c are the Argand diagrams of the computed impedance spectra *Z*(*ω*,*p*) of the fabric model under tension and compression, respectively. Figures 6b,d plot the changes in the magnitudes of the impedances as the amounts of the loadings increase.

We can observe interesting results from the Argand diagrams for both cases. Under tension, the real part of the impedance significantly changes with the amount of the loading, whereas the imaginary part shows negligible change for all chosen frequencies. Therefore, the changes of the impedance magnitude stemmed from the changes in the real part.

Under compression, both real and imaginary parts change with loading but the amounts of the changes are much smaller compared with the changes in the real part under tension. When we use the conductive fabric as a pressure sensor, we should expect more compression than tension. Therefore, it is necessary to measure both the real and imaginary parts of the impedance to estimate its changes associated with different amounts of loadings.

### 3.2. Experimental Results of EIS from Conductive Fabrics without Loading

**Figure 7.**
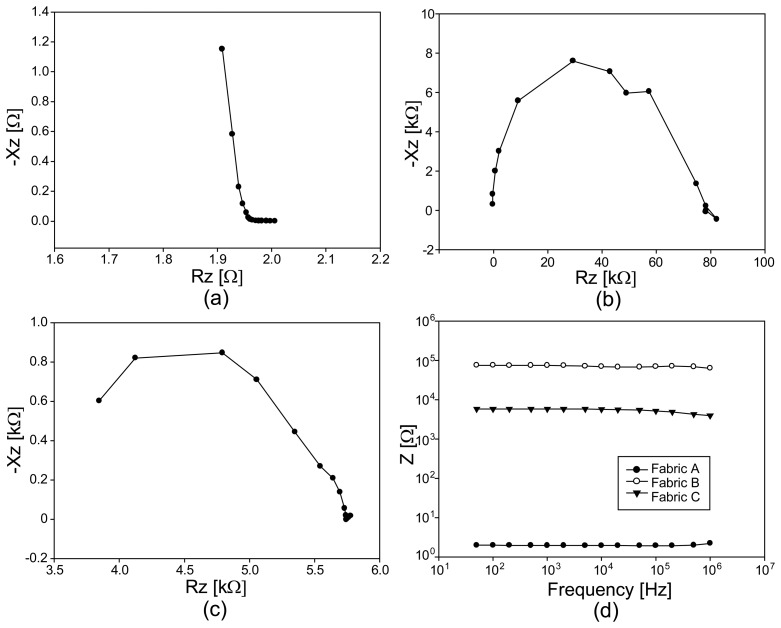
Argand diagrams of the impedance spectra from three conductive fabrics without any applied loading. (**a**), (**b**), and (**c**) are from the fabrics A, B, and C. (**d**) shows the plots of the impedance magnitude changes at 10 kHz.

Figure 7d shows the impedance magnitude spectra of the three chosen conductive fabrics, A, B and C. The impedance magnitudes were largest for the carbon-coated fabric B, which was least conductive. The most conductive silver-plated fabric A showed the smallest values for all chosen frequencies. To closely observe the effects of their compositions, we showed the Argand diagrams of the measured impedance spectra in Figures 7a–c. The highly conductive fabric A behaves more like a conductor at frequencies below 100 kHz with decreasing resistance (or real part) values. It exhibits a characteristic frequency of about 100 kHz where its imaginary part starts increasing.

Since the fabrics B and C have similar compositions, the difference in their Argand diagrams should be connected to their structural differences. The measured impedance values themselves were larger for fabric B since it was less conductive than fabric C. However, fabric B exhibits more capacitive effects, especially at high frequencies, due to its regularly weaved structure with many air gaps.

### 3.3. Experimental Results of EIS from Conductive Fabrics under Tension

**Figure 8.**
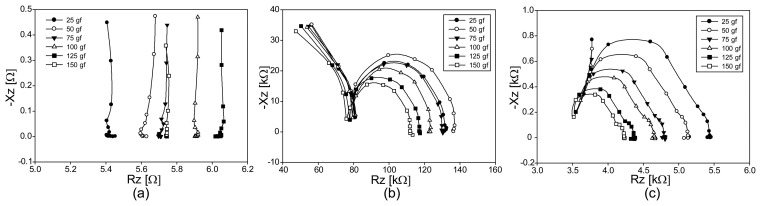
Argand diagrams of the impedance spectra from three conductive fabrics under tension. (**a**), (**b**), and (**c**) are from the fabrics A, B, and C, respectively.

We showed the Argand diagrams of the measured impedance spectra for fabrics A, B, and C under tension in Figures 8a–c, respectively. Since fabric A was highly stretchable, its Argand diagrams are similar to the ones from the numerical simulations in Figure 6a. The fabrics B and C were very stiff and their Argand diagrams indicate that they were not elongated by the applied tension. Instead, the axially applied loading by weights compressed the fabrics. Therefore, the Argand diagrams of the fabrics B and C show the patterns of the compression in Figure 6c. The high-frequency behaviors in Figures 8b,c are not visible in Figure 6c since we used only one parallel RC model in the numerical simulations.

**Figure 9.**
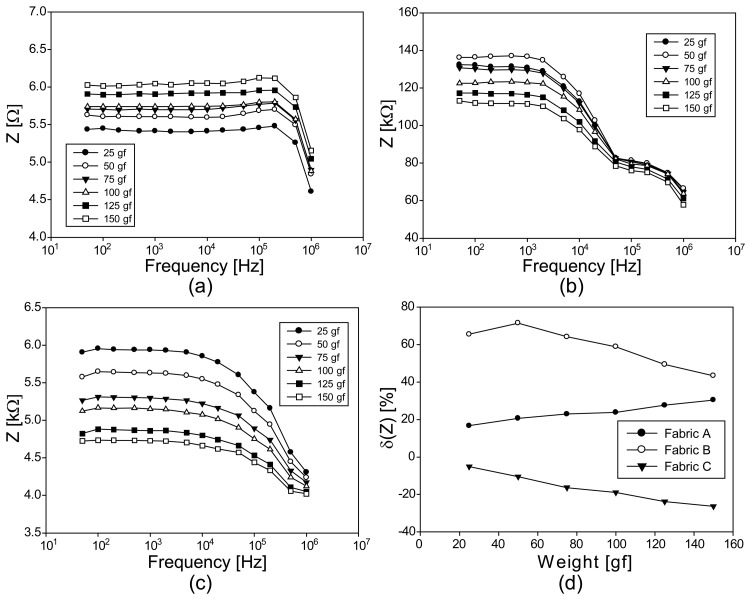
Impedance magnitude spectra of three conductive fabrics under tension. (**a**), (**b**), and (**c**) are from the fabrics A, B, and C, respectively; (**d**) shows the plots of the impedance magnitude changes at 10 kHz.

Figures 9a–c are the plots of the impedance magnitude spectra for fabrics A, B, and C, respectively, under seemingly applied tension. For the highly conductive fabric A, the plots are flat up to 100 kHz since their time constants RCs were small.

Comparing the impedance magnitude spectra of the fabrics B and C, fabric B seems to have a higher capacitive term. Comparing the Argand diagram in Figure 9a with those in b and c, we can observe that the impedance values change in the opposite directions as we increased the amount of tensile loadings. This is more clearly depicted in Figure 9d at 10 kHz. As mentioned in the previous paragraph, this indicates that the axially applied loading on fabrics B and C did not elongate the fabrics and behaved as compression in effect. The amount of the effective compression must have been smaller than the vertical compression described in the next section.

### 3.4. Experimental Results of EIS from Conductive Fabrics under Compression

**Figure 10.**
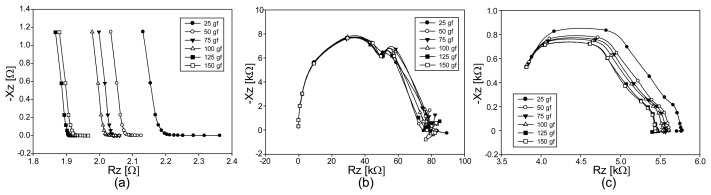
Argand diagrams of the impedance spectra from three conductive fabrics under compression. (**a**), (**b**), and (**c**) are from the fabrics A, B, and C, respectively.

We show the Argand diagrams of the measured impedance spectra for fabrics A, B, and C under compression in Figures 10a–c, respectively. For the stretchable fabric A, the changes of the impedance values are in the opposite direction compared with the case of tension in Figures 8 and 9. Though the least conductive fabric B shows large values of the measured impedance spectra, they do not change much subject to the applied compression. On the other hand, fabric C produces larger amounts of changes in the impedance spectra for different amounts of the applied compression.

**Figure 11.**
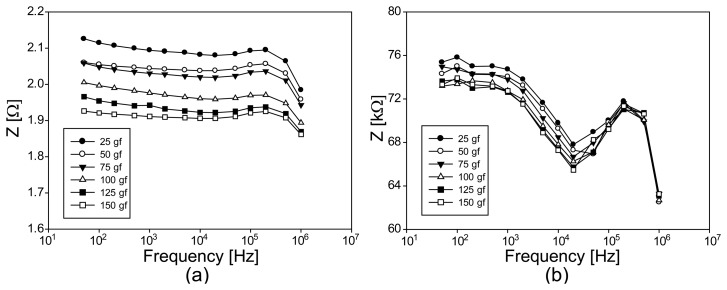

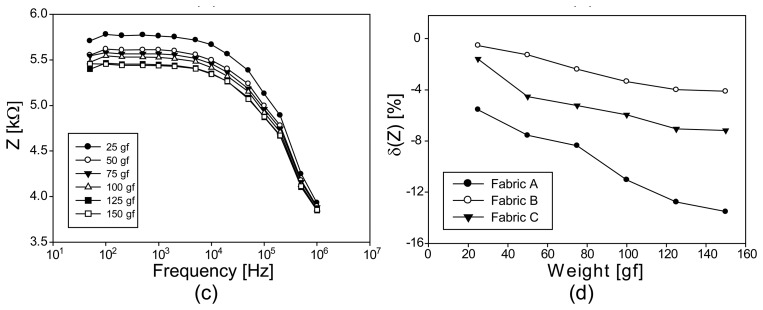
Impedance magnitude spectra of three conductive fabrics under compression. (**a**), (**b**), and (**c**) are from the fabrics A, B, and C, respectively; (**d**) shows the plots of the impedance magnitude changes at 10 kHz.

Figures 11a–c are the plots of the impedance magnitude spectra for fabrics A, B, and C, respectively, under applied compression. Unlike the results in Figure 9, all plots in Figure 11 show the changes in the same direction of compression. From the plots in Figure 11d, we can see that fabric A has the largest sensitivity to the applied compression. Therefore, if we consider the relative impedance changes only, fabric A appears to be the best candidate as a pressure sensor. Since the highly conductive fabric A produces small changes in its absolute impedance values, however, we will need to amplify the signals using a low-noise amplifier. Both fabrics B and C produce large impedance changes subject to the applied compression. However, fabric C appears to be better than fabric B since it has a higher sensitivity.

## 4. Discussion

Conductive fabrics are being widely used in biomedicine, robotics, and other industrial applications. The electrical impedance of a conductive fabric is determined by the electrical properties of its components and also its structure. Changes in the impedance stem from structural deformations subject to mechanical loadings such as tension or compression. We could evaluate the electromechanical behavior of a conductive fabric using the electrical impedance spectroscopy method and observe the effects of such structural deformations through its impedance spectrum.

With an applied tensile force, the fabric fibers were stretched and became thinner. Decreasing the fiber width, the fibers moved away from each other. This decreased the contact areas between the fibers and hence the impedance increased. On the other hand, under compression, the fabric fibers were compressed and flattened. This increased the fiber width and thus the fibers moved closer to each other to increase the contact areas among them. Hence, the area of the air gaps decreased and the impedance decreased.

From the numerical simulations and experimental results of the impedance spectra, we could characterize the electromechanical properties of the chosen three conductive fabrics. We suggest using the proposed methods to evaluate a fabric material as a candidate of a pressure sensor.

In the EIS of a conductive fabric, we can inject a constant current and measure the induced voltage. The impedance is determined not only by the material properties (conductivity and permittivity) but also by the geometry (shape and size) of the sample. Longer or wider fabric samples of the same material will have larger or smaller impedance values, respectively, even though their material properties are the same. Therefore, in practice, we should carefully control the current amplitude not to make the induced voltage out of its operating range.

For a given fabric sensor with a certain geometrical design, its impedance at no loading condition is predetermined. Without loading, fabric A has a smaller impedance value than those of fabrics B and C. However, fabric A shows a larger fractional change of the impedance with loading. By choosing a proper amount of injection current into fabric A, the fractional change of the induced voltage will also be larger since the voltage change is proportional to the impedance change subject to the applied pressure. It is desirable for a conductive fabric to produce large fractional impedance changes for a given range of loading. If we use a voltage amplifier with an enough signal-to-noise ratio (SNR), therefore, fabric A will have a higher sensitivity expressed in volt per Newton. If the SNR is not enough, fabrics B and C can be advantageous since they produce larger voltage signals for the same current.

To measure both tension and compression, we need to use a stretchable fabric such as fabric A. The fabrics B and C can be used only for compression. These characteristics stem from their fabrication methods. Since we used commercial fabric samples in this paper, we could not alter their compositions and fabrication methods such as coating, weaving, and density control. As previously mentioned, all of these should affect their electromechanical properties. We may consider a design problem where we specify a desirable electromechanical property such as a pressure sensor and fabricate such a conductive fiber. If we control the fabrication methods in future studies, we should be able to separately evaluate the effects of them including composition, coating, weaving, and so on.

We plan to develop an imaging system, which quantitatively visualize the pressure distribution on a sheet of a conductive fabric. We will install multiple electrodes around the boundary of the fabric sheet to inject currents and measure induced voltages. Expanding the EIS device to a multi-channel measurement system, we can collect boundary current-voltage data subject to many different current injection patterns. Adopting the image reconstruction methods of electrical impedance tomography (EIT) in biomedical applications, we plan to produce pressure images [45,46]. For this research goal, it would be useful to develop more sophisticated simulation methods including distributed parameter models and finite element models.

Noting that the electrical impedance of a conductive fabric changes with frequency, we may consider using the fabric together with a multi-frequency EIT system. Though we limited the maximum operating frequency as 1 MHz in this paper, future EIS studies at higher frequencies may reveal more electromechanical properties of the conductive fabrics.

## 5. Conclusions

We investigated how the electrical impedance spectrum of a conductive fabric changes subject to structural deformations under tension or compression using the electrical impedance spectroscopy (EIS) method. We found that the electrical impedance spectrum depends on the composition and structure of the given fabric. Under tension or compression, its electromechanical behavior can be captured as changes in its impedance spectrum mainly due to structural deformations. This means that conductive fabrics are potentially useful as pressure sensors for various applications. Since tensile and compressive loadings affect the impedance differently, we may separately measure those using stretchable conductive fabrics. We suggest testing various conductive fabrics using the EIS method to design a sensor to measure a pressure or visualize a pressure distribution as an image.
